# Oxime Esters as Efficient Initiators in Photopolymerization Processes

**DOI:** 10.3390/molecules31010187

**Published:** 2026-01-04

**Authors:** Monika Dzwonkowska-Zarzycka, Alicja Balcerak-Woźniak, Janina Kabatc-Borcz

**Affiliations:** Department of Organic Chemistry, Faculty of Chemical Technology and Engineering, Bydgoszcz University of Science and Technology, Seminaryjna 3, 85-326 Bydgoszcz, Poland; alicja.balcerak@pbs.edu.pl

**Keywords:** oximes, oxime esters, synthesis type I photoinitiator, photopolymerization

## Abstract

The development of new photoinitiators for photocurable systems has gained increasing interest in response to regulatory and environmental requirements, including efficient absorption in the UV/Vis range and reduced toxicity. Among emerging light-sensitive compounds, oxime esters have attracted growing attention as efficient radical photoinitiators. In this paper, five series of oxime esters based on carbazole, coumarin, carbazole–coumarin, phenothiazine, and triphenylamine scaffolds were described. Their high performance in photopolymerization processes was presented, demonstrating their ability to act as both type I and type II photoinitiators, as confirmed by high monomer conversion degrees. These data highlight oxime esters as versatile photoinitiating systems and provide a basis for further structural optimization aimed at improving water solubility and enabling comprehensive cytotoxicity assessment.

## 1. Characteristics of the Group

Oximes are a group of compounds with the general formula R_1_R_2_C=NOH, where R_1_ and R_2_ may be a hydrogen, alkyl, cycloalkyl or aromatic group. A characteristic feature is the presence of oxygen combined with strongly electronegative nitrogen. The precursor to the formation of the N-O bond is hydroxylamine (NH_2_OH) [1]. Among oxime derivatives, 7 classes of chemical compounds can be distinguished: oxime ethers, dioxime oxalates, oxime carbamates, oxime esters, oxime carbonates, oxime oxalate amides and oxime glyoxylates [2].

A significant group of these compounds are oxime esters. Oxime esters were first developed by Tranid in 1963. Since then, the relevance of this group of compounds has been constantly growing, expanding the state of knowledge and arousing greater interest among scientists [3,4]. The great potential of oxime esters stems from their biological properties, including antifungal, herbicidal, insecticidal, antitumor, and antibacterial activities [5,6,7,8,9].

Due to the unique properties of oxime esters, certain compounds have been introduced for commercial use. These include methomyl (*S*-methyl *N*-[(methylcarbamoyl)oxy]thioacetimidate), characterized by a broad spectrum of action against insecticides [10,11], and phoxim (*O*,*O*-diethyl *O*-(α-cyanobenzylideneamino) phosphorothionate), also used as an insecticide against lepidopteran pests [12].

**Figure 1:**
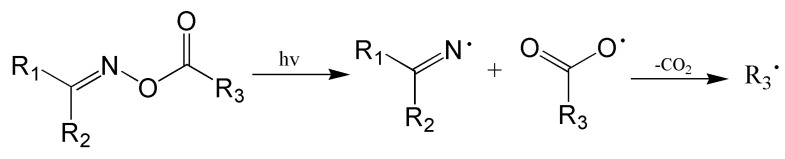
Fragmentation of the N-O bond. Reproduced with permission from [15], ChemPhotoChem; published by John Wiley and Sons, 2025.

Recently, scientific papers have highlighted the photoactive properties of these compounds. For several years, increased interest in this group of compounds in polymer chemistry and photochemistry has been observed. Oxime esters are promising candidates for initiating photopolymerization. Upon irradiation with a specific wavelength absorbed by the oxime ester, it undergoes excitation. Subsequently, the N-O bond cleavage occurs, which leads to the radical formation: iminyl and acyloxy [13]. The subsequent process—decarboxylation—results in the formation of not only CO_2_ but also a radical that can initiate photopolymerization (see Figure 1) [14].

It should be noted that low N-O bond dissociation energy (40–50 kcal·mol^−1^) [16,17], and, above all, generation of CO_2_, which prevents oxygen inhibition [15] and high rate of the reaction (due to the low requirements needed for the homolytic cleavage of N-O bond) [18] are a significant factors for effective photoinitiation of polymerization.

In addition to the above advantages, publications also highlight further benefits of oxime esters and explain the growing interest in this group of organic compounds. We are talking here about:

(1) Their potential for use in material jetting technology has been investigated. This technology offers many advantages (for example, high printing resolution), but the main problem is oxygen inhibition. The publication not only proved the possibility of using oxime esters, but also obtained (by studying the kinetics of photopolymerization) higher monomer conversion rates, FC = 80% for Omnirad 1316 compared to the commercially used BAPO (phenylbis(2,4,6-trimethylbenzoyl)phosphine oxide), where the monomer conversion rate was at the level of 60% [19].
(2) Numerous studies have confirmed the use of various chromophoric groups and the possibility of easily obtaining oxime esters sensitive to visible light, which is particularly important in ecological systems [20,21].
(3) From the perspective of photopolymerization, the degree of monomer conversion itself is also significant. Many studies demonstrate that oxime esters enable high monomer conversion.

Although several oxime esters are already commercially available, all absorb below the light visible region. Examples of oxime esters used as commercially available photoinitiators are listed in Table 1 [14,22,23,24,25].

## 2. Application of Oxime Esters as Photoinitiators

The process by which a liquid monomer or oligomer is converted into a solid material under the influence of light of a specific wavelength is called photopolymerization [26,27]. Due to its many advantages, which include the ability to work at room temperatures [28,29], use light as a source of energy (which makes this process energy-efficient), reduced use of often harmful organic solvents [30,31,32], spatial and temporal control of the process [33] or high rate of reaction [29], photopolymerization is gaining in importance. It is reflected in its increasing use in processes such as 3D printing [34], coatings [35], inks [36], dentistry [37], solar cells [38], and many others [39,40,41,42,43,44,45,46,47,48,49].

A key component in a light-sensitive composition is a photoinitiator. Its main task is to convert physical energy (provided as light) into chemical energy by decomposing appropriate active centers (cations, anions, or radicals) that initiate the photopolymerization process [50,51,52]. Based on the mechanism of radical formation after exposure to light, the following types of photoinitiators can be distinguished:

- Type I, which decays directly upon absorption of light. Radical pairs are generated through a highly efficient α-cleavage process;
- Type II, which decays in the presence of a co-initiator that is a donor of a proton or an electron and consists of two or more components [51,53,54,55].

Current trends in polymer chemistry focus on the search for photoinitiators that meet the following requirements: an absorption spectrum compatible with commercially available light sources, good thermal stability, and a high quantum yield. Additional requirements, such as non-toxicity to cells or good water solubility, are dictated by further use in the medical field [56]. The fact that it is easy and safe to synthesize is also significant. Considering the above, it seems reasonable to investigate oxime esters. What is important here is the relative ease of maneuvering the chromophore group and the lack of need to introduce additional ingredients, which frequently have adverse effects on physical health. Therefore, it is justified to search for type I compounds [20].

This paper focuses on explaining the phenomenon of oxime esters, their implementation, and systematizing the achievements to date in polymer chemistry, and, in summary, concludes with a characteristic of their photoactive properties.

In short, oxime esters consist of a functional oxime group linked to a substituent by an ester bond, giving a molecule that can absorb light [57]. The position of a substituent in oxime esters plays a special function. The R_1_ substituent (see Figure 1) is responsible for the light-absorbing capacity. Hence, appropriate chromophoric groups sensitive to light at specific wavelengths, e.g., carbazole [58] and coumarin [59], have been introduced at this position. Introducing a substituent at the R_2_ position of the oxime ester can also affect absorption properties and photoreactivity. Due to steric hindrance, introducing lower-molecular-weight substituents is more reasonable to improve photoreactivity. Lu’s group studied the role of the hydrogen atom and the methyl group in this position. It was found that the hydrogen substituent at the R_2_ position enhances the photoreactivity of the oxime ester [60]. R_3_ substituents affect the activation energy of the N-O bond cleavage and, at a later stage, the degree of conversion of monomer [15]. In view of the above, most studies have focused on the design, synthesis, and effects of substituents R_1_ and R_3_. The progress of the work is discussed in the next part of this paper.

### 2.1. Carbazole-Based Oxime Esters

Carbazoles are a broad group of compounds commonly used in photolymerization processes. It is related to their good thermal stability, availability, affordability, and ease of chemical modification [61].

The first paper focused on carbazole-based oxime esters and was published in 2004 by Ohwa and co-workers. The authors proposed OXE-02 ester as a highly active compound. Due to its characteristic features, such as sensitivity in compositions with black pigments, this compound was quickly commercialized by manufacturers of colour filter resist [14].

In 2011, Nie and co-workers also investigated oxime esters [62]. By introducing a chromophore group (based on a carbazole or diphenyl sulfide skeleton), four photoinitiators were obtained. These modifications were aimed at enhancing solubility and the significant red shift in the absorption spectrum.

**Figure 2:**
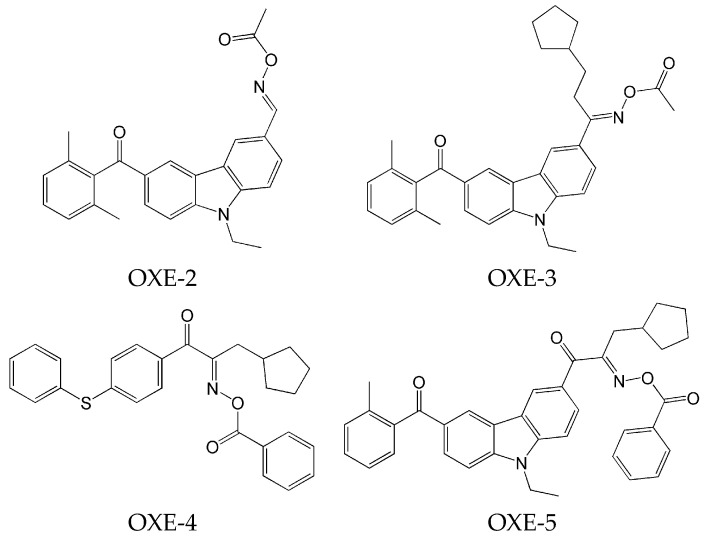
The chemical structures of the obtained compound. Reproduced with permission from [62], Journal of Applied Polymer Science; published by John Wiley and Sons, 2012.

Chemical structures of these compounds are presented in Figure 2.

All oxime esters described above were highly soluble in polar and nonpolar solvents and monomers (PTGDA—tripropylene glycol diacrylate and TMPTA—trimethylolpropane triacrylate). Additionally, OXE-3, OXE-4 and OXE-5 demonstrated high solubility in PGMEA (propylene glycol monomethyl ether acetate). These compounds are absorbed in the range between 300 nm and 400 nm.

The ability of oxime esters to initiate photopolymerization was assessed using RT-FTIR. The influence of four factors that could have a significant impact on this process was investigated. The following features were assessed:

- the effect of various photoinitiators on polymerization. The use of OXE-3, OXE-4, and OXE-5 results in higher HDDA conversion (96%) compared to OXE-2, where the conversion was approximately 90%;
- the effect of the concentration of photoinitiator on polymerization. It was tested at five concentrations of OXE-4 (0.1 wt%, 0.3 wt%, 0.5 wt%, 0.8 wt%, and 1.0 wt%). The optimal concentration was determined to be 0.5 wt%. Increasing the concentration of this photoinitiator to this level increased the polymerization rate. Above this point, the rate of polymerization began to decrease;
- the effect of light intensity on polymerization. It was determined by study of the influence of four light intensities (10 mW·cm^−2^, 30 mW·cm^−2^, 50 mW·cm^−2^, and 80 mW·cm^−2^) on the photoinitiating ability of OXE-3 with a concentration of 0.3 wt%. An increase in light intensity from 10 to 50 mW·cm^−2^ resulted in a threefold increase in the polymerization rate. At a light intensity of 80 mW·cm^−2^, both the rate of polymerization and conversion slightly decreased;
- the effect of the type of monomers on polymerization. It was evaluated by photopolymerization of monomers (HDDA, TPGDA, TMPTA, and TMPTMA) in the presence of OXE-5 at a concentration of 0.3 wt%. Higher conversion rates were observed with difunctional monomers.

**Figure 3:**
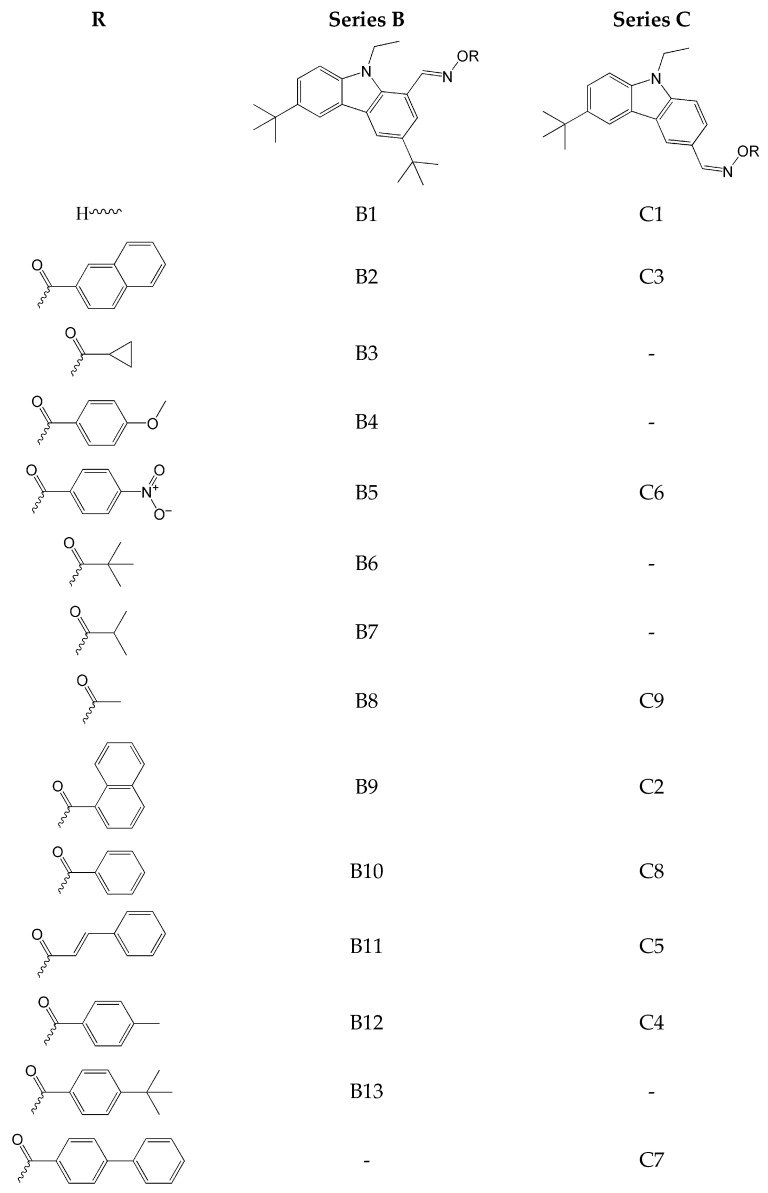
Chemical structures of tested compounds. Based on [63].

An interesting work describing effective photoinitiators based on carbazole skeleton was presented in 2023 by Feng and co-workers. The paper aimed to identify photoinitiators sensitive to light in the near-UV/Visible range [63]. The authors proposed 22 types of carbazole-based oxime esters, whose chemical structures are shown in Figure 3. Novel compounds were different in the number of *tert*-butyl groups and the type of photocleavable group attached to the carbazole moiety (series B and C). The introduction of *tert*-butyl groups increased the solubility of these compounds. All oxime esters were highly soluble in acetonitrile.

All of the obtained oxime esters exhibited a broad absorption band ranging from 320 to 400–450 nm. The maximum absorption for these compounds was observed in the UV-Vis region of the spectrum, at 360–382 nm for series B and 319–340 nm for series C, respectively.

The above-mentioned compounds were used to initiate photopolymerization under LED irradiation at 405 nm. The conversion of trimethylolpropane triacrylate (TMPTA) was monitored by RT-FTIR. It was found that the oxime esters of series B were more effective than those of series C. Additionally, those with an alkyl substituent showed the highest final monomer conversion, i.e., about 70% (for B3, B6, B7, and B8). Steady-state photolysis measurements showed that B7 and B8 photolysis intensify after 10 s of light exposure. Further prolongation of the process (beyond 60 s) does not yield favourable outcomes, and after this time, photolysis weakens. Based on ΔH_cleavage_ calculations, it was determined that the N-O bond cleavage occurs from the excited singlet state. The RT-FTIR technique was also used to study the decarboxylation reaction. A characteristic peak at 2337 cm^−1^ (after 60 s) was visible for most OXE/TMPTA formulations. Simultaneously, two of the best initiators (B7 and B8) were used in ESR-RT experiments to visualize the formation of radicals and confirm the occurrence of N-O bond cleavage. It was found that the acetyloxy radical and ethyl radical (for B7) and the acetyloxy radical for B8 were found. Based on these results, a reaction mechanism was proposed.

The final stage was to determine the feasibility of applying the B8/TMPTA system to direct laser writing (DLW) and achieve high spatial resolution within 2 min [63].

**Figure 4:**
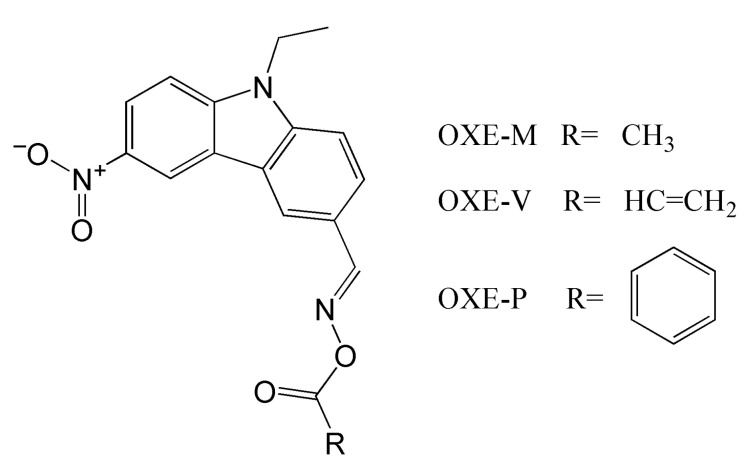
Chemical structures of OXE-(M,V,P). Based on [64].

Another group of carbazole-based compounds utilized in photopolymerization experiments is presented in Figure 4 [64]. The research group synthesized four compounds: three with an oxime ester moiety and one with an oxime group (OXE-H).

These compounds absorb UV-Vis light. The molar extinction coefficient of these OXEs is summarized in Table 2.

Steady-state photolysis confirmed the decay of these compounds upon LED@405 nm irradiation. The RT-FTIR measurements showed the formation of CO_2_ during photopolymerization. The FC values obtained for TMPTA were 69%, 57%, and 61% for OXE-M, OXE-V, and OXE-P, respectively. These results were similar to those when TPO was used as a photoinitiator (FC = 65%). The improved values observed for OXE-M photoinitiator may be due to the presence of a methyl substituent, which creates a smaller spherical barrier. Due to its most desirable results, OXE-M was chosen to test the suitability for laser writing and 3D printing. Differential scanning calorimetry (DSC) was used to assess thermal stability and thermal-initiating ability. The obtained results allowed the selection of OXE-M as advantageous in dual photo-thermal curing (decomposition by 113 °C). The assumption was confirmed when the TMPTA formulation was combined with carbon fibres, yielding a fully cured polymer.

To elucidate the relationship between the chemical structure and the photoinitiating ability of oxime esters, further studies were conducted [65]. A series of fifty oxime esters based on the nitro-carbazole skeleton was synthesized. This combination of structures (nitro and carbazole) resulted in good light-absorption properties in the visible range, up to 440 nm. Additionally, the molar extinction coefficient at 405 nm confirmed that all oxime esters can be irradiated with a diode emitting at this wavelength. The steady-state photolysis performed resulted in a decrease in absorbance intensity over irradiation time for λ_max_. A shift of the maximum of absorption towards red light was also observed, which was explained by cleavage of the N-O bond. In order to determine the favourability of the cleavage reactions, enthalpies of the cleavage process for the N-O bond were calculated. The results obtained suggested the favourable occurrence of the decay process from the singlet state (ΔH_cleavage s1_ < 0). However, subsequent results did not rule out the possibility of cleavage from the T1 state. Based on these, a photochemical mechanism of oxime esters was proposed.

**Figure 5:**
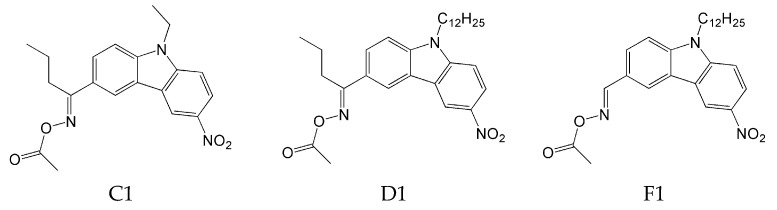
The chemical structure of the obtained oxime esters with the best conversion results. Reproduced with permission from [65], Macromolecules; published by ACS, 2022.

The photoinitiation ability of OXEs was also determined using RT-FTIR. TMPTA was used as a model monomer. The experiment proved that:

- The introduction of an oxime-ester group affects photoinitiation performance. Compounds without the oxime-ester group showed low photoinitiation ability. For example, compounds with the -OH group exhibited final conversion of monomer (FC) from 23% to 38%, in comparison to compositions with oxime esters, where FC values ranged from 38% to 68%;
- the type of substituent in OXE affects the degree of monomer conversion. Compounds with a methyl group demonstrated better photoinitiation ability compared to other compounds of the same series. The use of C1, D1, or F1 results in higher TMPTA conversion than with commercially available TPO. The chemical structures of the oxime esters with the best conversion results are shown in Figure 5.

- the increase in concentration of oxime ester results in an increase in the final conversion of TMPTA.

The authors also explained the influence of substituent type on photoinitiation ability. The light-absorption properties of all oxime esters were similar and could not serve as a basis for these considerations. The explanation was sought in the decarboxylation process. Theoretical calculations showed that oxime esters with methyl substituents are highly reactive (ΔH_decarboxylation_ = −4.94 kcal/mol for methyl group, for benzoyloxy or acryloyloxy groups: ΔH_decarboxylation_ > 0 kcal/mol). The research group observed a correlation between the time of CO_2_ generation during the decarboxylation reaction and the monomer conversion. Between 10 s and 16 s of irradiation, the acrylate function increased rapidly, which was related to CO_2_ generation. When CO_2_ generation stopped at 16 s, conversion was almost complete. However, both the absorbance intensity of CO_2_ and the final conversion of monomer were higher for oxime esters with methyl substituents. Based on the results, the D1/TMPTA system was selected, and direct laser writing (DLW) and 3D-printing experiments were conducted. High-resolution objects were achieved. In addition, several systems were selected for thermal behaviour testing. Three of them, namely B1, C1, and D1, proved to be suitable for dual photo/thermal initiator behaviour. The research conducted was significant in that it proved and explained the importance of decarboxylation in the final conversion of monomers and paved the way for further research into the design of high-performance oxime ester-based photoinitiators.

The presented examples demonstrate the feasibility of incorporating oxime ester functionality into carbazoles and their use in light-induced processes. The combination of carbazoles and oxime esters may contribute to their even wider use as type I photoinitiators.

### 2.2. Coumarin-Based Oxime Esters

**Figure 6:**
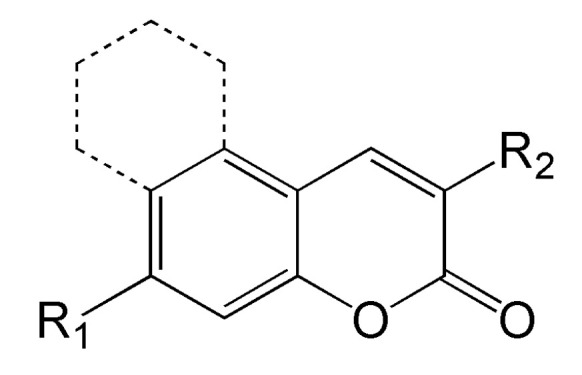
Possible modification of coumarins.

Widely used photoinitiators include coumarins. It is related to their advantages, such as:

- the option of being called bio-sourced molecules. Substances of natural origin have recently been gaining increasing interest [66];
- the chemical structure that allows a wide range of modification. Figure 6 depicts the chemical structure of coumarins. Possible modification allows on, i.e., (1) in position R_1_ for tuning of the solubility, and (2) in position R_2_, also tuning of the solubility and introduction of the type I moiety [67];
- The possibility of using them as both type I and type II photoinitiators.

The main advantage of type I photoinitiators is that they do not require additional substances (such as amines, which can have a negative impact on health), thereby enhancing their relevance in the medical industry. Additionally, this may have a positive impact on the economy.

An experiment conducted in 2018 [68] was focused on the design of unimolecular systems based on a coumarin skeleton. An additional advantage of the compounds obtained was their sensitivity to visible light and their ability to photobleach. Most compounds that absorb in the visible-blue light range are yellow, which limits their use in applications where colourless compositions are important (e.g., dental materials). The ability to photobleach can overcome this feature, as photobleachable photoinitiators lose their colour after light exposure.

**Figure 7:**
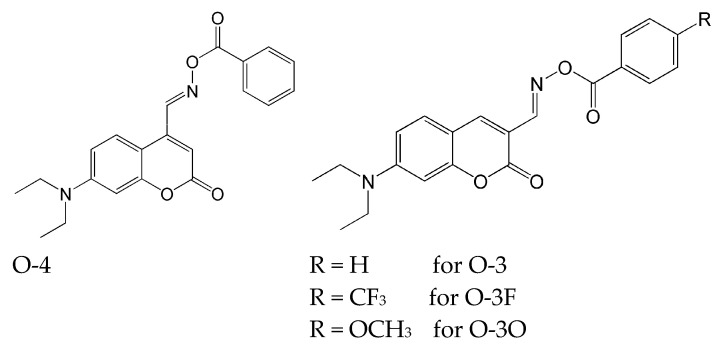
The chemical structure of compounds based on the coumarin molecule. Reproduced with permission from [68], Applied Materials; published by ACS Publications, 2018.

The chemical structures of the synthesized compounds are shown in Figure 7.

These compounds exhibited a broad absorption band spanning 400–480 nm. Interestingly, when comparing compounds O-3 and O-4, the change in the position of the substituent in coumarin had no significant impact on the maximum absorption band (as further proven by theoretical HOMO-LUMO calculations). However, this change influenced ε_max_ (for O-4 ε_max_ = 7680 M^−1^ cm^−1^, for O-3 ε_max_ = 41 690 M^−1^ cm^−1^) and fluorescence emission (for O-4 λ_em_ (nm) was 596 and for O-3 λ_em_ (nm) was 500). It is also worth mentioning fluorescence quantum yields, which should be low for type I photoinitiators, since it promotes the presence of active states to produce active free radicals for subsequent polymerization. The steady-state photolysis process varied depending on the type of substituent:

- O-4: the initial exposure to the light caused a slight shift of the maximum of absorption to red light, and the absorbance increased. Further irradiation causes the absorption maximum to shift slightly to blue light, and a slight decrease in the corresponding absorption was observed.
- O-3, O-3F and O-3O: where the maximum absorption decreased with the light exposure. After 10 min, a loss of yellow colour was observed.

As mentioned above, a distinguishing feature of oxime esters is their ability to generate CO_2_ as a product of decomposition. To detect its presence, a phenolphthalein solution was used in combination with K_2_CO_3_, which was connected to the O-3 solution via a tube. The experiment showed a loss of colour in the phenolphthalein from pink to colourless after 10 min of irradiation, which confirmed the release of CO_2_.

**Figure 8:**
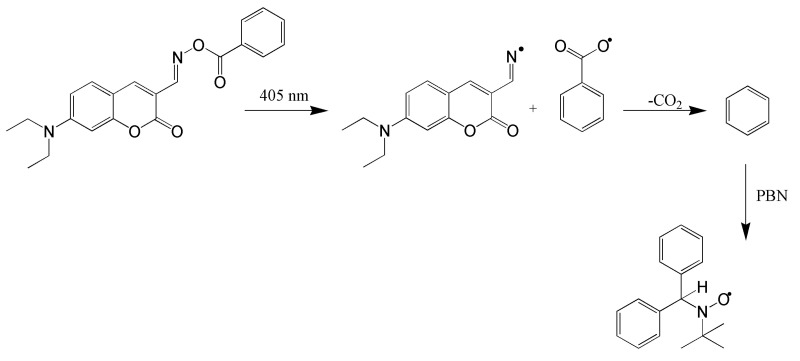
Mechanism of photoinitiator decomposition. Reproduced with permission from [68], Applied Materials; published by ACS, 2018. PBN—phenyl-*N*-*tert*-butyl-nitrone.

Sequentially, the ESR experiment confirmed the formation of phenyl radicals. The authors’ proposed mechanism for photoinduced decomposition is shown in Figure 8.

**Figure 9:**
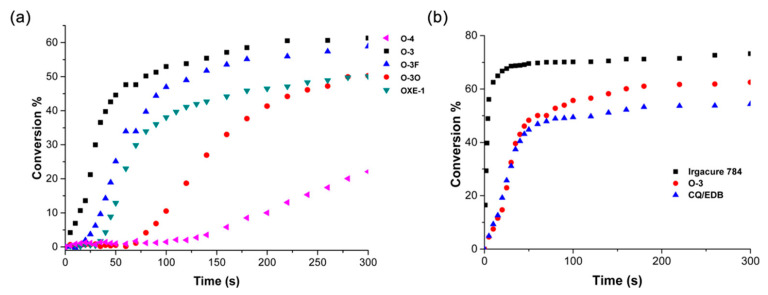
Double-bond conversion with various (**a**) oxime ester PIs and (**b**) commercial visible light PIs (system) concentration: (5.5 × 10^−5^ mol/g resin), irradiated by 450 nm visible LED light with an intensity of 200 mW·cm^−2^. Reproduced with permission from [68], Applied Materials; published by ACS, 2018.

The novel photoinitiators were tested in photopolymerization experiments using LED@450 nm as the light source (intensity of 200 mW·cm^−2^). The process kinetics were monitored in real time using FTIR spectroscopy. The double-bond conversion during photopolymerization time is shown in Figure 9.

Next, an oxime ester designated O-3 was synthesized and compared with commercially available photoinitiators—Irgacure 784 and CQ/EDB. O-3 proved to be superior to the CQ/EDB system commonly used in dental materials.

The same O-3 compound effectively induced thiol-ene polymerization. The reaction was completed in practically 20 s with the following conversion results: PETMP/TAIC (Pentaerythritol tetrakis(3-mercaptopropionate)/triallyl isocyanurate) and PETMP/TAC (triallyl cyanurate) up to 80% and up to 90% conversion for thiol and vinyl double conversion, respectively. For compositions with PETMP/APE (pentaerythritol triallyl ether), S-H conversion up to 80% and the vinyl double bond conversion up to 95%.

Photobleaching studies confirmed the whitening properties of the thiol-ene resin composition of the O-3 photoinitiator system. After 2 min of light exposure, the irradiated surface lost its yellow colour. Further, after 10 min of exposure, the bleached surface was 2.6 mm thick (total sample thickness was 4.8 mm).

Thermal stability tests were also performed in the experiment. In the absence of monomer, oxime ester O-3 showed a lower decomposition temperature than commercially known OXE-1 (150 °C and 185 °C, respectively). In the presence of monomer, the decomposition temperature was 70 °C for OXE-1 and 106 °C for O-3.

In summary, the experiment proved the possibility of using coumarin in combination with oxime esters as type I photoinitiators.

The aforementioned possibility of using oxime esters based on the coumarin skeleton as type I and II photoinitiators was also described by Lalevée’s group in 2021 [16]. Ten compounds with different substituents were synthesized and used in photopolymerization experiments.

**Figure 10:**
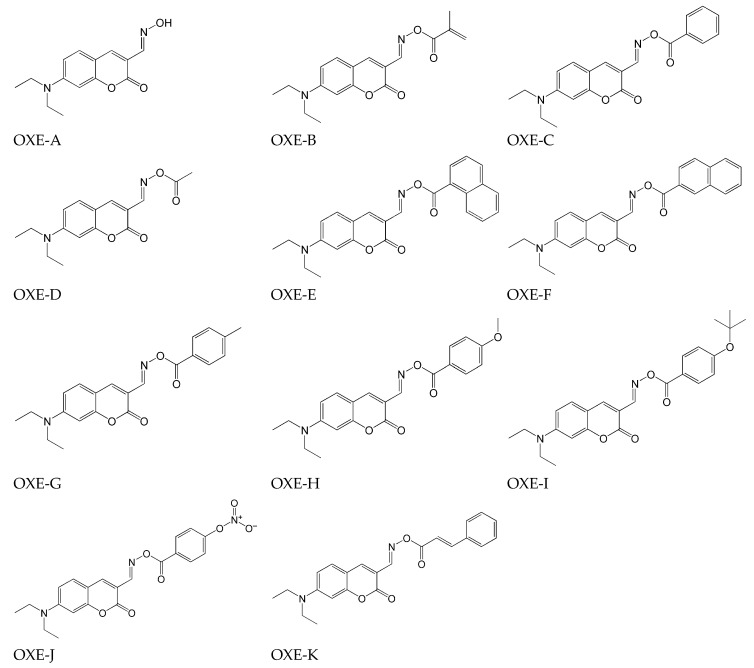
Chemical structures of tested compounds. Based on [16].

The chemical structures of the tested compounds are presented in Figure 10.

Due to the presence of the coumarin moiety, all molecules absorbed in the visible light region with an absorption maximum ranging from 418 nm (OXE-A without an ester group) to 441 nm (OXE-J with a nitro-phenyl substituent). The spectroscopic properties confirmed the compatibility of the absorption range with LED@405 nm. The introduction of oxime-ester group functionality caused a red-shift of λ_max_ compared to the OXE-A compound (with N-OH group, where λ_max_ = 418 nm) and an increase in the molar extinction coefficient. The use in photopolymerization was tested with TMPTA as the monomer and the photoinitiator at 0.5% wt. In two-component systems, Iod (1% w) was used as an additional co-initiator. The reaction conditions were room temperature, irradiation: LED@405 nm, light intensity I_0_ = 110 mW/cm^2^. The kinetics results are summarized in Table 3.

In both cases (one- and two-component systems), the use of OXE-J (with nitro-phenyl substituent) leads to the highest final conversion (FC%) of TMPTA, which is related to its highest absorption properties. A compound with a similar degree of monomer conversion is OXE-D (methyl substituent), for which no direct correlation between conversion and absorption was observed. However, it is related to the decarboxylation process, as confirmed by calculations and the value of H_dcarboxylation_ < 0 (i.e., −4.94 kcal/mol). Hence, the radicals (R) formed need to be more effective for initiating the polymerization reaction. The higher reaction rate observed with using two-component systems, unrelated to the absorption properties, is explained by their photochemical reactivity with the iodonium salts. It was confirmed by steady-state photolysis. The photolysis of oxime esters alone occurred more slowly than in the presence of iodonium salts, showing that the direct cleavage of OXEs is less favourable than their interaction with iodonium salts. Thermal-initiation ability tests using DSC showed excellent properties (T_onset_ = 155 °C, T_max_ = 181 °C) and confirmed the possibility of use in both thermal and photopolymerization processes.

The coumarin skeleton allows a wide range of modifications, facilitating its development and improvement as useful photoinitiators. Endowing coumarin with oxime ester functionality may further increase interest in this group of organic compounds as initiators of type I photopolymerization. This approach may result in development towards “Green Chemistry”.

### 2.3. Carbazole-Coumarin-Based Oxime Esters

**Figure 11:**
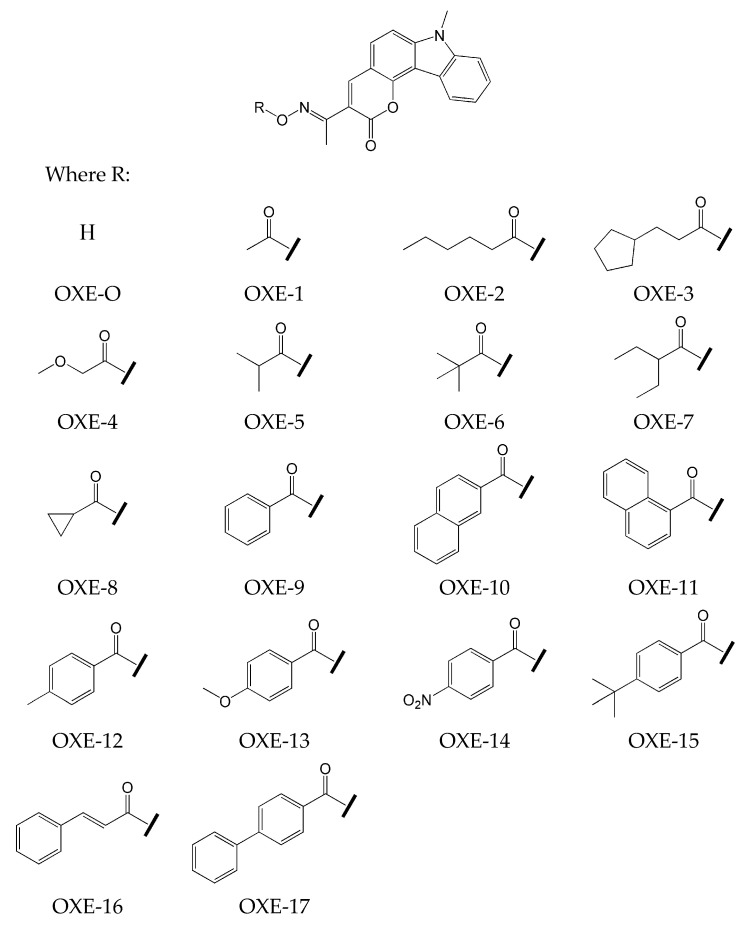
The chemical structure of the obtained compounds. Based on [69].

Based on previous results, Zhang’s research team [69] decided to combine these compounds and prepared carbazole-fused coumarin-based oxime esters. The seventeen new compounds were synthesized. The chemical structures of these compounds are presented in Figure 11.

The introduction of an oxime ester group into the chemical structure results in a bathochromic shift of λ_max_ compared to OXE-0 (without the functionality of the oxime ester group). Steady-state photolysis resulted in decomposition and a decrease in absorbance. A rapid decrease was observed after 10 s of irradiation.

Based on the photopolymerization experiment monitored by real-time FT-IR, it was found that:

- oxime esters OXE-1, OXE-2, OXE-5, and OXE-7 showed higher conversion of monomer (about 67%) than the commercially available photoinitiator TPO (FC = 63%) (TMPTA) (concentration of photoinitiator was 2 × 10^−5^ mol/g).
- the highest rates of polymerization were achieved for OXE-1, OXE-2, OXE-5, OXE-7, and OXE-12, where the values of R_p_/[M_0_] × 100 were 5.6 s^−1^, 6.9 s^−1^, 5.9 s^−1^, 6.5 s^−1^, and 8.9 s^−1^, respectively.

The presence of CO_2_ was also monitored using RT-FTIR (absorbance peak at 2337 cm^−1^). A characteristic peak was observed for OXE-1-OXE-6 and OXE-8. The ESR-ST experiments conducted for OXE-1 and OXE-5 confirmed the formation of acetyl and isobutyryl radicals. The above-mentioned oxime esters were used as photoinitiators for the polymerization of TMPTA in DLW. The objects formed by polymerization of the OXE-5/TMPTA and OXE-1/TMPTA systems showed good resolution within a short time (approx. 1 min).

To confirm OXEs’ ability to generate radicals, DSC analysis was performed. OXE-1 and OXE-5 oxime esters showed dual photo/thermal initiator behaviour. Their T_initial_ (88.9 °C and 111.6 °C) and lower T_max_ (179.2 °C and 181.5 °C) values were sufficient to consider them for the preparation of carbon-fibre composites. The experiment was conducted to investigate the activation of oxime esters under sunlight. The samples (thick about 1.4 mm) were evaluated using the IR technique. The obtained conversions were as follows:

- for OXE-1 with TMPTA—15.4%; with Ebecryl605 52%;
- For OXE-5 with TMPTA—9.2%; with Ebecryl605 44.5%.

Despite the low conversions obtained with TMPTA formulations, this experiment demonstrates that sunlight can serve as a light source for generating radicals from oxime esters. This study may serve as a basis for expanding the prospects for the use of oxime esters.

The combination of different chromophore groups may be an excellent response to the growing demand for light-sensitive initiators active in the Vis range.

### 2.4. Phenothiazine-Based Oxime Esters

The use of phenothiazines as chromophores has excellent potential, as they absorb in the 315–400 nm range. Familiarity with this fact facilitates the design of hotoinitiators compatible with the visible range of light [70]. Additionally, it has an excellent electron-donating group that exhibits additional properties, e.g., antimicrobial, which expands its scope of application [14]. They are often used as type I and II photoinitiator systems.

**Figure 12:**
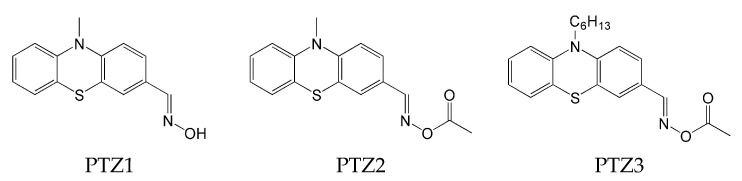
The chemical structures of phenothiazine-based oxime esters. Based on [70].

The phenothiazine skeleton served as the basis for another experiment aimed at studying the photoinitiating properties of oxime esters in mono- and multicomponent systems [70]. The chemical structures of proposed compounds are shown in Figure 12.

A spectroscopic study confirmed that the introduction of an ester-oxime group shifts the absorption maximum to longer wavelengths. Compound PTZ1, which contains an -NOH group, has a λ_max_ at 325 nm. The λ_max_ values for PTZ2 and PTZ3 shift to 345 nm and 355 nm, respectively. The highest molar absorption coefficient was calculated for PTZ3 (ε_max_ = 6600 M^−1^ cm^−1^, and ε_405_ = 2600 M^−1^ cm^−1^). The obtained compounds showed a broad absorption band (up to 500 nm) and compatibility with a visible light source.

The ability to initiate photopolymerization using the proposed type I compounds as photoinitiators was tested using TA (di(trimethylolpropane)tetraacrylate) as a monomer by RT-FTIR. A 405 nm LED was used as the light source. The final acrylate conversions are presented in Table 4.

The conducted experiment confirmed the effectiveness of the presence of the oxime-ester group in the compound, and its favourable effect on the light-induced polymerization process was confirmed.

**Figure 13:**
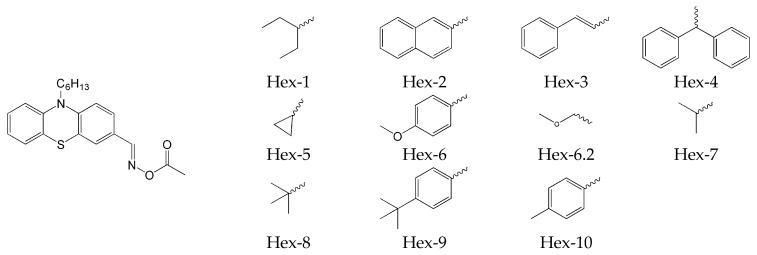
The chemical structures of the compound based on the PTZ3 structure. Based on [70].

It should be emphasized that this process was also carried out for a wide range of substituents introduced on the carboxyl side (Hex-1–Hex-10) of the PTZ3 structure. The chemical structures of phenothiazine-based oxime esters are presented in Figure 13.

All compounds showed lower conversion rates than PTZ3. The highest conversion rate achieved by Hex-10 was approximately 75%.

**Figure 14:**
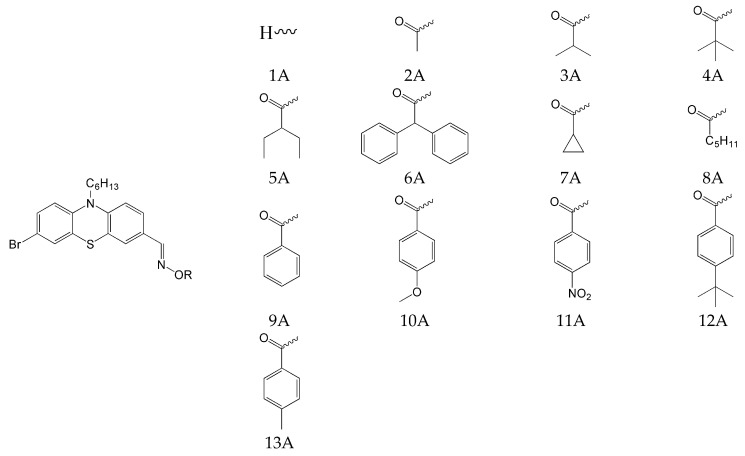
The chemical structures of compounds based on the PTZ3 (with heavy atom bromide) structure. Based on [70].

The effect of incorporating a heavy atom, bromide, into phenothiazine was also considered. The chemical structures of the studied compounds are presented in Figure 14.

**Figure 15:**
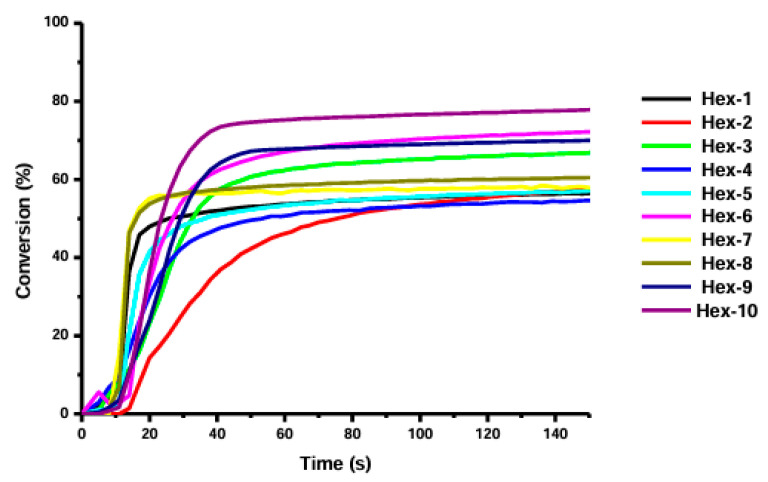
Photopolymerization profiles of TA in laminate (thickness = 25 μm) upon exposure to LED (λ = 405 nm) initiated by PIs (0.5% *w*/*w*). Based on [70].

**Figure 16:**
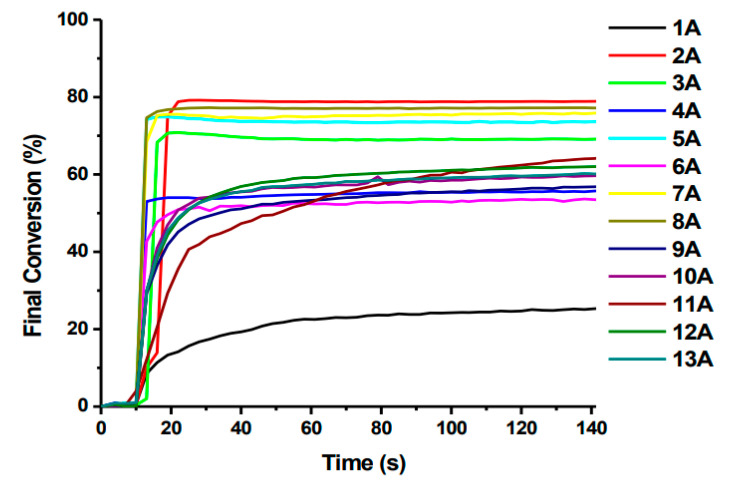
Photopolymerization profiles of TA in laminate (thickness = 25 μm) upon exposure to LED (λ = 405 nm) initiated by PIs (0.5% *w*/*w*). Based on [70].

It was confirmed that introducing a bromide atom improves the conversion rate. Among compounds 1A–13A, five oxime esters showed conversions of 70–80%. In the case of compounds Hex1-Hex-13, only two showed conversion in this range. The progress of the photopolymerization process is presented in Figure 15 and Figure 16.

The practical applicability was tested using the DLW technique (source: laser diode @405 nm) with PTZ3. The obtained 3D patterns exhibited high thickness and high spatial resolution.

Additionally, the main structures (PTZ1-3) were subjected to their thermal initiator features. The obtained results (Table 5) confirmed the possibility of using PTZ2 and PTZ3 in an industrial application.

As mentioned earlier, phenothiazines find applications in multi-component photoinitiating systems. These properties were checked with respect to Iod at a dye/Iod concentration (1%/1% *w*/*w*) as a photoinitiating system for TA monomer polymerization upon irradiation with LED@405 nm. The obtained results are summarized in Table 6.

It was found that the presence of phenothiazine derivatives (PTZ1–PTZ3) significantly improves the photopolymerization process. This increase is most noticeable for the PTZ1 and PTZ2 compounds, where the mono- to two-component conversion increases from 14% to 68% for PTZ1 and from 71% to 82% for PTZ2 during free radical polymerization of TA. The evaluation of the ability to initiate cationic polymerization with respect to EPOX resulted in a conversion rate of 83% for PTZ2.

In 2023, Lalevée and co-workers synthesized 12 compounds based on the phenothiazine molecular skeleton with double oxime-ester functionality [71]. The chemical structures of the mentioned compounds are presented in Table 7.

All obtained compounds exhibited bands ranging from 475 nm, with maximum absorption for all compounds being similar (between 362 nm and 375 nm). The introduction of oxime-ester groups resulted in a red shift of the absorption maximum.

The steady-state photolysis experiment proved:

- Absence of decay of compounds and no decrease in absorbance intensity under the influence of light @405 nm for compounds: OXE-A0 and OXE-B0 (with -NOH group);
- generally, oxime esters undergo photolysis when exposed to light at a wavelength of 405 nm. Additionally, for OXE-B2 and OXE-B4, an increase in absorbance intensity was observed at a wavelength of 350 nm, which is explained by the formation of a new product.

The ability to photoinitiation the polymerization of TMPTA by oxime esters was investigated and compared with the commercially available photoinitiator: TPO. Oxime esters of the A series showed higher FC than those of the B series. The use of OXE-A1, OXE-B2 and OXE-B4 led to higher FC values of TMPTA (80%, 74% and 66%) than TPO (65%). Additionally, such high conversion rates were achieved at very low concentrations (i.e., 1 × 10^−5^ mol·g^−1^) or from 0.45 wt% to 0.76 wt%. An additional conclusion from the study was the determination of the importance of the distance between chromophores and degradable groups. A larger distance may contribute to the occurrence of higher FCs in the monomer formulation—OXE-A1 80% and OXE-B1 55%. Compounds with the highest FC (%) showed rapid decay after 10 s exposure to light. This work is significant because it confirms the possibility of initiating the photopolymerization process with double oxime esters. It opens new possibilities for designing more efficient photoinitiators.

The advantages of phenothiazines, which undoubtedly include absorption in the range of 315–400 nm, make them popular molecules in the field of initiators. The conducted studies confirmed their applicability as both type I and type II photoinitiators.

### 2.5. Triphenylamine-Based Oxime Esters

A group of compounds that should be mentioned are triphenylamine derivatives. Its wide use in polymer chemistry is due to certain significant advantages:

- ease of structural modification, it is also possible to easily adjust the absorption band to the entire visible range,
- ease of purification of triphenylamine derivatives,
- good solubility in most organic solvents,
- triphenylamine is readily available and cheap [72].

**Figure 17:**
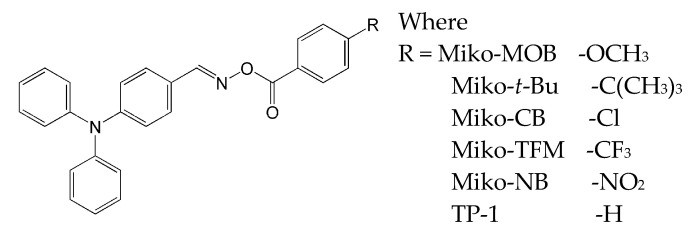
The chemical structures of the studied compounds. Reproduced with permission from [15], ChemPhotoChem; published by John Wiley and Sons, 2024.

A particular focus was the impact of terminal moieties on photoreactivity. To investigate this phenomenon, five oxime esters based on the triphenylamine molecule were synthesized, where the electron-donating (-OCH_3_, -C(CH_3_)_3_) and electron-withdrawing (-Cl, -CF_3_, -among NO_2_) substituents on the carbonyl side were benzene derivatives [15]. The results were compared with the compound containing a benzene ring without the additional substituent. The chemical structures of the studied compounds are presented in Figure 17.

The spectroscopic properties revealed no significant influence of various benzene substituents on electron delocalization, as evidenced by a similar absorption maximum around 360 nm. Only in the oxime ester with the nitro group (Miko-NB), the absorption spectrum shifted towards the red end of the spectrum. Interestingly, all newly formed oxime esters exhibited a higher molar extinction coefficient than the reference compound (TP-1).

The thermal properties of oxime esters, studied by TGA (thermogravimetric analysis) and DSC (differential scanning calorimetry), indicated the need for high temperatures for decomposition. It was found the following order of thermal stability: Miko-NB (191 °C) > Miko-MOB (186 °C) > Miko-CB (178 °C) > Miko-*t*-Bu (177 °C) > Miko-TFM (165 °C). The reduction potential: it can be seen that the values for compounds with electron-withdrawing substituents (Cl or CF_3_) are lower than those for compounds with electron-donating substituents. This phenomenon was explained by electron affinity, which increases after the addition of halogen groups, leading to a lower reduction potential. All mentioned electro-optical and thermal parameters are presented in Table 8.

The photolysis experiments were performed on two compounds (Miko-MOB and Miko-NB) using an LED at 365 nm. A faster photochemical decomposition process was observed during irradiation at 365 nm, due to a higher molar extinction coefficient at this wavelength and a greater ability to absorb energy and to cleave bonds. It should also be noted that Miko-NB showed a very low photolysis rate at 365 nm and did not decompose when exposed to 405 nm. The ESR analysis confirmed the formation of radicals resulting from the decomposition of the N-O bond.

Photo-DSC was used to study the kinetics of free-radical photopolymerization. The following parameters were determined: the maximum polymerization rate, the time at maximum heat flow, and the conversion of TMPTA.

Considering irradiation with a wavelength of 405 nm, the parameters such, as: maximum rate of polymerization and the final double bond conversion were, as follows: Miko-MOB (0.79 s^−1^, 32%) > Miko-t-Bu (0.68 s^−1^, 30%) > TP-1 (0.48 s^−1^, 23%) > Miko-TFM (0.28 s^−1^, 24%) > Miko-CB (0.22 s^−1^, 21%) > Miko-NB (0.03 s^−1^, 3%). The results obtained under radiation at 365 nm were also similar and showed a similar trend. Interestingly, the results obtained are significant in that they demonstrate the influence of substituent groups on the benzene ring on photolysis, photoreactivity, ESR, and thermal and optical properties. The results obtained indicate the possibility of modifying organic compounds to obtain oxime esters with the required photoreactivity and adequate storage stability.

**Figure 18:**
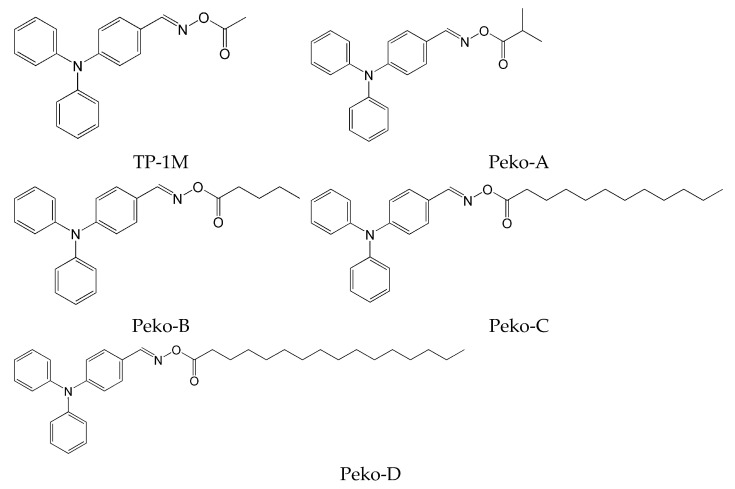
The chemical structures of the studied compounds used in the experiments. Reproduced with permission from [73], ChemistrySelect; published by John Wiley and Sons, 2023.

The influence of peripheral substituents on the initiation ability of free radical photopolymerization in triphenylamine was described by Hsieh and co-workers [73]. It was shown that the methyl substituent is more reactive than the benzene substituent in oxime esters [74]. The literature reports that different alkyl substituents can alter light-absorption properties. The three-step synthesis yielded five oxime esters bearing the triphenylamine skeleton. The chemical structures of the studied compounds are shown in Figure 18.

All described oxime esters exhibited an absorption range from 250 nm to 400 nm, with two maxima at about 290 nm and 350 nm. The elongation of the alkyl substituent increased the molar absorption coefficient. The lowest value was for the oxime ester with a methyl substituent (TP-1M), and the highest for the pentadecane substituent (Peko-D). The electro-optical parameters, thermal properties, bond dissociation energies, and triplet-state energies of the compounds are presented in Table 9.

The analysis of the fluorescence spectra showed high emission intensity, which may reduce the efficiency of radical generation. However, this ability is influenced by other factors (such as light-harvesting or bond-dissociation energy), hence the need for further study. Thermogravimetric analysis revealed low decomposition temperatures (T_d_), which are justified by the presence of N-O bonds, which are relatively easy to cleave. The compound to mention here is Peko-D, which had the highest T_d_. It also had the highest melting point. The ESR experiment performed for Peko-A and Peko-D revealed the same mechanism of active radical generation. In addition, the amount of radicals generated by Peko-A was higher, which may affect subsequent rapid photopolymerization.

These oxime esters undergo rapid photodegradation within the first 2 s upon exposure to light at 405 nm and 365 nm. Interestingly, further exposure to LED@365 nm did not cause any changes. At 405 nm, an increase in the short-wavelength region was observed.

**Figure 19:**
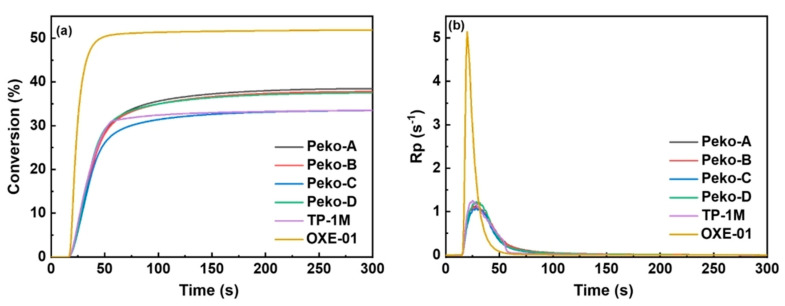
(**a**) Conversion versus time and (**b**) Rp versus time of TMPTA photopolymerization initiated by oxime esters under UV light irradiation in 50 mW·cm^−2^. Reproduced with permission from [73], ChemistrySelect; published by John Wiley and Sons, 2023.

**Figure 20:**
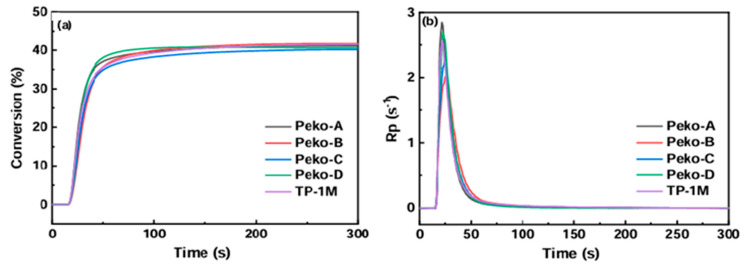
(**a**) Conversion versus time and (**b**) Rp versus time of TMPTA photopolymerization initiated by oxime esters under UV light irradiation in 180 mW·cm^−2^. The irradiation starts for t = 24 s (oxime esters/TMPTA (2/98) (wt%/wt%)). Reproduced with permission from [73], ChemistrySelect; published by John Wiley and Sons, 2023.

**Figure 21:**
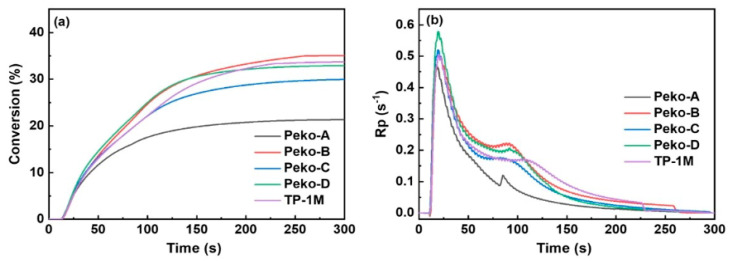
(**a**) Conversion versus time and (**b**) Rp versus time of TMPTA photopolymerization initiated by oxime esters under LED@365 nm light irradiation in 50 mW·cm^−2^. Reproduced with permission from [73], ChemistrySelect; published by John Wiley and Sons, 2023.

**Figure 22:**
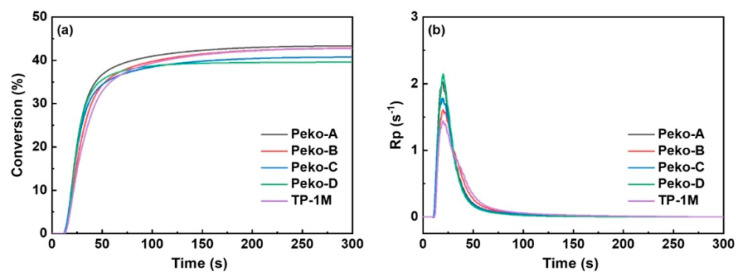
(**a**) Conversion versus time and (**b**) Rp versus time of TMPTA photopolymerization initiated by oxime esters under LED@405 nm light irradiation in 50 mW·cm^−2^. Reproduced with permission from [73], ChemistrySelect; published by John Wiley and Sons, 2023.

From the application’s perspective, a photopolymerization experiment was also performed using photo-DSC. All esters of the Peko series, TP-1M, and commercially available OXE-01 were subjected to the experiment. UV lamps (operating range λ = 250–450 nm, intensity = 50 or 180 mW·cm^−2^), LED@365 nm (intensity = 50 mW·cm^−2^) or LED@405 nm (intensity = 50 mW·cm^−2^) were used as light sources. The results obtained are presented in Figure 19, Figure 20, Figure 21 and Figure 22.

Considering the UV light source, it was shown that (1) higher light intensity results in higher final double bond conversions (40–41%), (2) Peko-A, Peko-B, and Peko-C oxime esters exhibit a wide range of activity at different light intensities.

Under the influence of LED@365 nm radiation, the efficiency of all oxime esters decreases (compared to UV lamps). The reason may be surface curing under shorter wavelength exposure conditions. Compared to LED@365 nm, photopolymerization using LED@405 nm resulted in higher conversion rates. In addition, better penetration was observed. These studies were interesting because they showed that to improve photopolymerization rate, it is necessary to properly assess the molar extinction coefficient and use an appropriate light source, thereby influencing the efficiency of radical generation.

## 3. Conclusions

A noticeable increase in interest in oxime esters across various industries has also occurred. As type I photoinitiators, they offer new possibilities for the research and development of photopolymerization processes. To meet the growing demand for initiators sensitive to Vis radiation, many chromophore groups have been studied (for example, carbazole, coumarin, phenothiazine, and triphenylamine). The substituents adjacent to the carbonyl group (α-position) are also subject to modification, intending to improve the following parameters: rate of process, the final conversion of monomer, and depth of cure. Further work should focus on optimizing existing photoinitiators to eliminate adverse effects (e.g., the filter effect), as well as on developing oxime esters for dual thermal/photochemical curing to improve curing depth. In addition, current activities also require work to be directed towards “Green Chemistry”. One of these assumptions is pertinent for obtaining water-soluble structures and assessing cytotoxicity. New directions for development may also be indicated by combining different chromophore groups, which can mutually offset their negative characteristics.

## Figures and Tables

**Table 1:** Commercially available oxime esters photoinitiators. Based on [14,22,23,24,25].

Chemical Structure	Abbreviation or Trade Name	Maximum Absorption, nm	Application
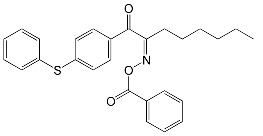	OXE-1 Omnirad 1314	330	UV-curable filter, resin formulation, electronics
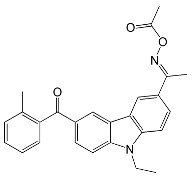	OXE-2 Irgacure	338	pigments, LED curing, electronics
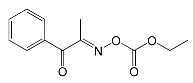	Speedcure PDO	259	electronics, adhe-sives, pigments
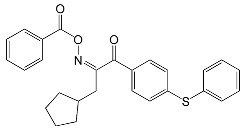	Speedcure 8001	240 and 331	pigments, LED curing
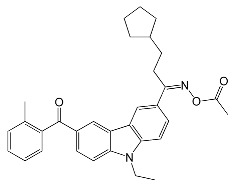	Speedcure 8002	340	pigments, LED curing, electronics

**Table 2:** Light absorption properties of tested compounds. Reproduced with permission from [64], Macromolecular Rapid Communications; published by John Wiley and Sons, 2021.

Compound	ε_max_ (M^−1^ cm^−1^)	Ε_405nm_ (M^−1^ cm^−1^)
OXE-P	13,800	4100
OXE-M	13,000	4100
OXE-V	12,400	3900

**Table 3:** Final acrylate function conversion (FCs) and the polymerization rate for TMPTA using one-component (1% wt) photoinitiators and two-component OXE/Iod (0.5%/1% *w*/*w*) photoinitiating systems. Based on [16].

PI	Final Conversion (FC) (%) One-Component	R_p_/[M]_0_ × 100 (s^−1^) One-Component	Final Conversion (FC) (%) Two-Component
OXE-A	34	1.14	71
OXE-B	55	5.17	63
OXE-C	60	1.16	73
OXE-D	72	6.03	78
OXE-E	52	1.22	56
OXE-F	61	2.39	65
OXE-G	59	1.58	71
OXE-H	42	1.05	66
OXE-I	56	0.99	57
OXE-J	73	5.61	79
OXE-K	54	1.09	59

**Table 4:** FCs of acrylate function using one-component photoinitiating system (1% *w*/*w*) after 150 s of irradiation with an LED (*λ* = 405 nm). Based on [70].

PIs	FC in Laminate LED@405 nm
TPO	83%
PTZ1	14%
PTZ2	71%
PTZ3	81%

**Table 5:** Maximal, onset polymerization temperatures and final acrylate FCs for TA using 1% *w*/*w* of PIs as thermal initiators (under N_2_ atmosphere). Based on [70].

PIs	T_onset_ [°C]	T_max_ [°C]	Conversion [%]
PTZ1	206	241	37
PTZ2	135	169	34
PTZ3	125	171	36

**Table 6:** FCs of acrylate (TA) and epoxy function using two-component photoinitiation system PI/Iod (1%/1% *w*/*w*) and Iod alone (1% *w*/*w*) after 150 s (in TA) and 800 s (in epoxy) of irradiation with a LED@405 nm. Based on [70].

PI	Conversion of TA [%]	Conversion of Epoxy [%]
PTZ1/Iod	68	81
PTZ1/Iod	82	83
PTZ2/Iod	83	69
Iod	23	38

**Table 7:** Chemical structures of tested compounds. Based on [71].

R	Series A	Series B
	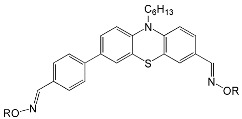	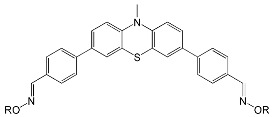
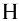	OXE-A0	OXE-B0
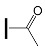	OXE-A1	OXE-B1
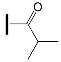	OXE-A2	OXE-B2
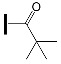	OXE-A3	OXE-B3
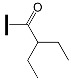	-	OXE-B4
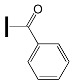	-	OXE-B5
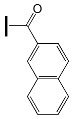	-	OXE-B6
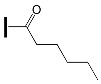	-	OXE-B7
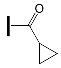	-	OXE-B8
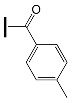	-	OXE-B9

**Table 8:** Electro-optical parameters and thermal properties of the compounds. Reproduced with permission from [15], ChemPhotoChem; published by John Wiley and Sons, 2024.

Sample	λ_abs_ (ε × 10^4^ M^−1^ cm^−1^) (nm) ^a^	λ_PL_ (nm) ^a^	E_ox_ (V) ^b^	E_red_ (V) ^c^	E_g_ (eV) ^d^	T_d_ (°C) ^e^	T_m_ (°C) ^f^
Miko-TFM	295 (2.23), 367 (5.25)	485	0.82	−1.36	3.07	165	N.D.
Miko-CB	295 (1.88), 364 (4.52)	467	0.81	−1.37	3.06	178	N.D.
Miko-NB	256 (2.73), 368 (3.06)	N.D.	0.84	−1.20	3.10	191	N.D.
Miko-MOB	263 (2.95), 361 (3.81)	466	0.82	−1.27	3.09	186	N.D.
Miko-*t*-Bu	296 (1.49), 361 (3.30)	465	0.85	−1.20	3.10	177	123
TP-1	294 (0.91), 360 (2.23)	467	0.90	−1.34	3.05	174	129

^a^ Recorded in DCM solutions at 10^−5^ M. ^b^ Oxidation potential. Recorded in CH_2_Cl_2_ solutions. Scan rate, 100 mV s^−1^, electrolyte, nBu_4_NPF_6_. ^c^ Reduction potential. Recorded in CH_2_Cl_2_ solutions. Scan rate, 100 mV s^−1^, electrolyte, nBu_4_NPF_6_. ^d^ The bandgap E_g_ was derived from the cut-off wavelength of the absorption spectra. ^e^ Temperatures at 5% weight loss recorded by TGA at a heating rate of 15 °C min^−1^ in N_2_. ^f^ Melting point.

**Table 9:** Electro-optical parameters, thermal properties, bond dissociation energy and triplet state energy. Reproduced with permission from [73], ChemistrySelect; published by John Wiley and Sons, 2023.

Sample	λ_abs_ [ε × 10^4^ M^−1^ cm^−1^] [nm] ^a^	λ_PL_ [nm] ^a^	E_ox_ [V] ^b^	E_red_ [V] ^c^	E_g_ [eV] ^d^	ΔG_ET_ [kJ mol^−1^] ^e^	T_d_ [°C] ^f^	T_m_ [°C] ^g^	BDE N-O [kcal mol^−1^] ^h^	E_T_ [kcal mol^−1^] ^i^
Peko-A	294 (1.68), 354 (3.13)	460	0.86	−1.28	3.13	−95.4	172	N.D.	48.21	53.87
Peko-B	294 (1.20), 356 (2.50)	467	0.57	−1.44	3.13	−107.9	177	67	48.87	53.89
Peko-C	294 (1.65), 354 (3.02)	460	0.73	−1.41	3.13	−95.4	183	64	48.83	53.89
Peko-D	294 (1.85), 354 (3.57)	461	0.80	−1.39	3.12	−89.6	209	76	48.83	53.89
TP-1M	294 (1.08), 356 (2.25)	-	0.57	−1.18	3.15	−134.8	-	-	48.75	53.89

^a^ Recorded in DCM solutions at 10^−5^ M. ^b^ Oxidation potential. Recorded in CH_2_Cl_2_ solutions. Scan rate, 100 mV s^−1^, electrolyte, nBu_4_NPF_6_. ^c^ Reduction potential. Recorded in CH_2_Cl_2_ solutions. Scan rate, 100 mV s^−1^, electrolyte, nBu_4_NPF_6_. ^d^ The bandgap E_g_ was derived from the cut-off wavelength of the absorption spectra. ^e^ Free energy change. ^f^ Temperatures at 5% weight loss recorded by TGA at a heating rate of 15 °C min^−1^ in N_2_. ^g^ Melting point. ^h^ N-O bond dissociation energy. ^i^ Triplet state energy.

## Data Availability

No new data were created or analyzed in this study. Data sharing is not applicable to this article.
